# Effects of a Smartphone App on Fruit and Vegetable Consumption Among Saudi Adolescents: Randomized Controlled Trial

**DOI:** 10.2196/43160

**Published:** 2023-02-09

**Authors:** Israa M Shatwan, Rabab S Alhefani, Mawadah F Bukhari, Danah A Hanbazazah, Jumanah K Srour, Shelini Surendran, Najlaa M Aljefree, Noha M Almoraie

**Affiliations:** 1 Food and Nutrition Department Human Sciences and Design Faculty King Abdulaziz University Jeddah Saudi Arabia; 2 Faculty of Health and Medical Sciences University of Surrey Guildford, Surrey United Kingdom

**Keywords:** smartphone app, fruit and vegetable consumption, Saudi Arabia, adolescents, nutrition, health outcome, digital health intervention, digital health app, pediatrics, youth

## Abstract

**Background:**

Dietary patterns and nutritional status during adolescence have a direct effect on future health outcomes.

**Objective:**

This study aimed to promote fruit and vegetable intake among adolescents using a smartphone app called MyPlate.

**Methods:**

This randomized intervention study was conducted in an urban area of Jeddah, Saudi Arabia. We included 104 adolescents aged 13 to 18 years, who were randomized into intervention (n=55) or control (n=49) arms. We examined the effects of MyPlate on fruit and vegetable intake over 6 weeks in the intervention group. Pre- and postintervention questionnaires were used in the intervention and control groups.

**Results:**

The control group showed a significant increase in fruit consumption scores between baseline (1.15, SD 0.68) and postintervention (1.64, SD 0.98; *P*=.01), but no significant difference in vegetable consumption scores was observed before (1.44, SD 0.97) and after intervention (1.55, SD 0.90; *P*=.54). However, there was no significant difference between scores at baseline and after 6 weeks of using the smartphone app for fruit (1.48, SD 0.99 and 1.70, SD 1.11, respectively; *P*=.31) or vegetables (1.50, SD 0.97 and 1.43, SD 1.03, respectively; *P*=.30) in the intervention group. Our findings showed no significant impact of using a smartphone app on fruit and vegetable consumption.

**Conclusions:**

These findings suggest that a smartphone app did not significantly improve fruit and vegetable intake among adolescents.

**Trial Registration:**

ClinicalTrials.gov NCT05692765; https://clinicaltrials.gov/ct2/show/NCT05692765

## Introduction

Adolescence is a critical period in the growth and development of an individual. Thus, dietary habits during this timeframe are important in shaping future health outcomes [1]. A large proportion of adolescents worldwide do not receive optimal nutrition [2]. Typically, adolescent diets are characterized by low fruit and vegetable (FV) intake and a high intake of energy-dense, nutrient-poor foods, including sugar-sweetened drinks and fast foods [3]. As a result, anemia and micronutrient deficiencies are common among adolescents, which have a significant effect on their quality of life [4]. The incidence of overweight and obesity among adolescents continues to increase worldwide and is associated with an increased risk for noncommunicable diseases, including hypertension, atherosclerosis, nonalcoholic fatty liver disease, and metabolic syndrome [5].

A diet rich in FVs is associated with numerous health benefits. FVs are a good source of vitamins A and C, minerals, electrolytes, phytochemicals, antioxidants, and dietary fiber [6]. The World Health Organization recommends FV intake amounting to ≥400 grams per day to maintain optimal health; reduce the risk of noncommunicable diseases, including heart disease, cancer, type II diabetes mellitus, and obesity; and prevent micronutrient deficiencies [6]. However, FV consumption among adolescents does not meet these recommendations. For instance, the UK National Diet and Nutrition Survey found that consumption of fruits during adolescence declined compared with early childhood, even though vegetable consumption showed no change [7]. In Saudi Arabia, most adolescents report that they consume low amounts of FVs daily [8,9]. Education and knowledge about food and nutrition are among the factors that influence food choices [6]. School-based nutrition education intervention targeting increased consumption of FVs has been shown to be an effective solution for low intake of FVs among children and adolescents. A 2-year nutrition intervention study showed a significant increase in FV consumption among children and their parents [10]. Using technology is one strategy to increase consumption of FVs among adolescents [11].

Recently, smartphone apps have been used to promote health and wellness among individuals in various communities. As a result, several nutritional interventions have been attempted to determine the usefulness of these apps in promoting healthy dietary habits. One of the advantages of these apps is that they can serve as cost-effective and flexible platforms for implementing behavioral changes in nutrition [12]. Additionally, mobile phone use is common across all education and income levels, and smartphone apps can serve as an appropriate platform to deliver healthy diet interventions, such as FV-related information, to adolescents and young adults [12]. Moreover, FV intake in adults and adolescents has been promoted by smartphone apps and web-based interventions [13,14]. The MyPlate software was developed by the US Department of Agriculture as a simple and powerful educational tool to aid individuals of different age groups in the appropriate distribution of food groups and creation of balanced meals according to the American Dietary Guidelines [15,16]. Brown et al [17] reported a significant increase in fruit consumption among an intervention group in comparison with a control group; the study used frequent text messages to provide nutrition-related knowledge and encouraged healthy behaviors among study participants. Likewise, Schroeter et al [18] demonstrated a significant increase in the intake of whole grains and FVs in an intervention group compared with a control group; the intervention group participated in MyPlate nutrition education meetings for 4 weeks, which positively affected their health behaviors.

In this study, we aimed to examine the effects of the MyPlate smartphone app on FV intake over 6 weeks in adolescents from Jeddah, Saudi Arabia.

## Methods

### Participants and Recruitment

**Figure 1:**
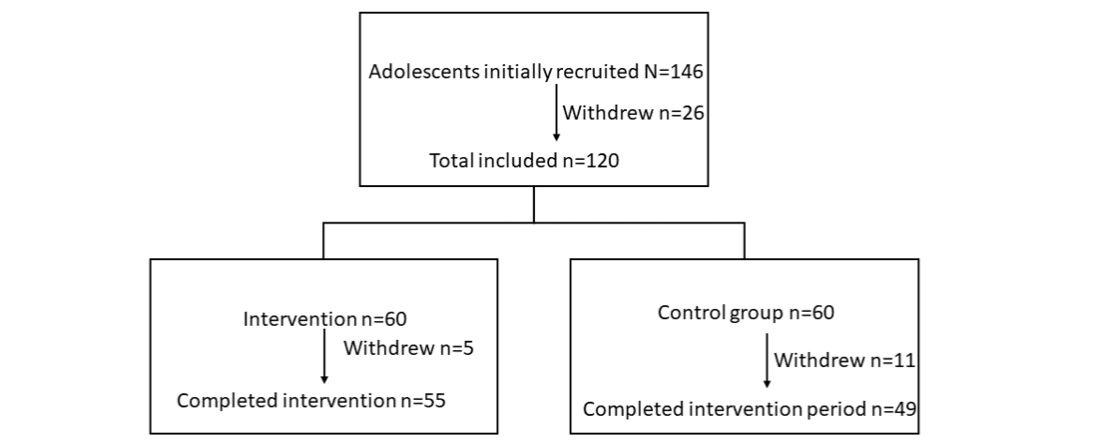
CONSORT (Consolidated Standards of Reporting Trials) diagram for the trial.

This randomized intervention study was conducted between February and March 2021 among adolescents from Jeddah, Saudi Arabia. Since schools were closed because of the COVID-19 pandemic, adolescents were recruited through invitations sent via email and WhatsApp to their parents using snowballing recruitment. The invitation to the study was first sent to members and bachelor of science students at the Food and Nutrition Department of King Abdulaziz University to help the research team with recruitment. Then, the research team contacted the parents and adolescents, who voluntarily agreed to participate in the study. The study procedures were explained to the parents or guardians of all prospective participants. There were no imposed circulation restrictions or curfews in Jeddah during the study period. Participants were included if they were in good health (based on the self-reported absence of diseases, such as diabetes mellitus, that may influence food intake), aged 13 to 18 years, attending school, and had access to a smartphone, either their own or their parents’. The exclusion criteria were poor health or an age not within the age range of this study. In total, 146 adolescents were initially recruited, of whom 26 withdrew from the study because they did not complete the baseline questionnaire or voluntarily decided to withdraw. The remaining 120 adolescents were randomly divided into intervention and control groups. Microsoft Excel (version 22, Microsoft Corp) with the *RAND* function was used to randomize the sample. After generating a random number, the participants were divided into control and intervention groups. As 16 adolescents decided not to complete the study or did not fill in the final questionnaire, 104 adolescents (24 adolescent boys and 80 adolescent girls) completed the intervention phase of the study (Figure 1).

The sample size was determined based on the ability to detect an expected mean difference of 0.7 servings, a value that was chosen based on a previous parallel intervention study [18,19], with 80% power and a 5% significance level. The calculated required sample size was 33 adolescents in each group [20]. Thus, anticipating a nearly 50% drop-out rate, we recruited 60 participants for each group.

### Ethics Approval

The study procedures were approved by the Unit of Biomedical Ethics Research Committee at King Abdulaziz University (101-21). All adolescents participated voluntarily and provided verbal consent for participation in the study; their parents or guardians provided written informed consent. This trial was registered on ClinicalTrials.gov (NCT05692765).

### Study Design

This was a randomized intervention study conducted with 104 adolescents: 49 in the control group and 55 in the intervention group. Adolescents in the intervention group were divided into 11 smaller groups, each containing 5 participants, to explain the app. One of the researchers conducted video conference calls with each of these small intervention groups. The researcher provided a brief presentation about the health benefits and appropriate serving sizes of FVs and explained how to use the smartphone app. An instructional brochure was provided in Arabic to all adolescents in the intervention group. The research team was available to assist participants at any time during the study period. The participants were required to choose 3 of 7 goals for fruits and 3 of 7 goals for vegetables. The fruit goals were as follows: (1) have fruit with dinner; (2) add fruit to your salad; (3) snack on fruit; (4) have fruit for a sweet treat; (5) have fruit with lunch; (6) add frozen, canned, or dried fruit to your meal; and (7) start your day with fruit. The vegetable goals were as follows: (1) have vegetables with dinner; (2) have a dark green vegetable; (3) start your day with vegetables; (4) have a red or orange vegetable; (5) have vegetables with lunch; (6) snack on vegetables; and (7) make a salad or side dish using beans, peas, or lentils. After choosing 6 goals (3 for fruit and 3 for vegetables), the participants were required to mark the goal that they chose daily and were requested to adhere to their chosen goals until the end of the study. They were encouraged to turn on notifications for the app to receive reminder messages. The research team also sent weekly WhatsApp text message reminders (in Arabic) to the adolescents. The intervention period was 6 weeks. Adolescents in the control group were not exposed to the smartphone app and did not receive any advice to promote their FV consumption, which may have affected their FV consumption. Instead, they were only asked to complete the pre- and postintervention questionnaires.

This study used the free nutritional MyPlate smartphone app, which is readily available on both iOS and Android platforms, to promote FV intake among adolescents. The app uses a multicomponent communications plan that was developed by the US Department of Agriculture Food and Nutrition Service in 2011. The app aids in translating the American Dietary Guidelines to the public and can be used as a nutritional education resource for children and adults. The app icon is an easy, effective, visual platform that helps promote healthy food choices that include all food groups and create a balanced plate at mealtimes. The app allows one to set daily healthy eating goals for each food group and track individual progress [15,16]. Additionally, the Saudi dietary guidelines (including Healthy Saudi Plate) have been developed based on evidence from several dietary guidelines, including the American dietary guidelines [21]. Since no Arabic-language apps are available in smartphone stores with similar features, we used the MyPlate app in the current study.

### Data Collection

All measures were collected via an online questionnaire on Google Forms. The questionnaire consisted of two parts. The first part included sociodemographic data, including information regarding age, sex, school type (private or public), weight and height of the adolescent and their parents, the parents’ education level (high school or lower, bachelor’s degree, or postgraduate degree), the parents’ employment status (employed or unemployed), number of children in the family, and family income. This part of the questionnaire was completed by the participant with the assistance of one of their parents. The research team provided instructions to participants on the appropriate way to measure height and weight using a weight scale and measuring tape. BMI was calculated as the weight in kilograms divided by the height in meters squared (kg/m^2^). BMI was evaluated using the Saudi growth chart (BMI for age). A BMI between the 15th and 85th percentiles was considered normal; BMI between the 85th and 95th percentiles was considered overweight, and BMI above the 95th percentile indicated obesity [22].

### FV Consumption Questionnaires

A validated food-frequency questionnaire (FFQ) was used to compare FV consumption in adolescents at baseline and after the intervention period in both groups. The FFQ was one that has previously been used with some adaptations to make it suitable for Saudi adolescents [13,18]. The response for each FV item was recorded as 1 of 7 frequency options (never, 1-3 times per month, 1 or 2 times per week, 3-4 times per week, 5-6 times per week, once per day, or 2 or more times per day). In total, 22 FFQ items were directly related to vegetable intake, whereas 18 FFQ items were directly related to fruit intake (Multimedia Appendix 1 shows the FVs listed in the FFQ). The response for each FV item had 7 frequency options; thus, it was scored based on a 7-level scale. The highest score was 7 for 2 or more per day, and the lowest score was zero (ie, never). The FV scores were calculated separately, and the sum of the scores for all fruit items for each participant was divided by the total number of fruit items in the FFQ (18); a similar process was used for vegetable scores.

### Data Analysis

Descriptive and inferential statistics were calculated using SPSS for Windows (version 27.0; IBM Corp). Frequency analysis was conducted to evaluate the baseline sociodemographic characteristics of the sample. The differences in age and BMI for adolescents and parents at baseline between the two study groups were compared using a 2-tailed *t* test. Changes in FV scores between baseline and after the intervention period in the control and intervention groups were analyzed using univariate regression. The smartphone app’s effectiveness was examined by comparing FV scores between the intervention and control groups at 6 weeks through univariate regression. Univariate linear models were adjusted for age, sex, parental education, family income, adolescent and parental BMI, and baseline values in the analysis to test the effects of the smartphone app. The data are reported as means (SD). A *P* value <.05 was considered statistically significant.

## Results

### Demographic Characteristics of the Study Sample

In total, 104 adolescents completed the study, of whom 23.1% were boys (24/104). The mean age of the adolescents was 15.1 (SD 1.6) years, and 75% (78/104) attended public school. The mean body weight was 56.9 (SD 15.8) kg. Most of the adolescents were in the normal body weight range, whereas 10.6% (13/104) were overweight, and 4.8% (6/104) were obese (Table 1). The majority of parents held a bachelor’s degree as their highest level of education. Most fathers were employed, whereas most mothers were unemployed. Most families had a middle to high income level, defined as a household income of 10,000 to 20,000 SR (US $2666 to $5333). The proportion of boys was lower in the intervention group. No other significant baseline differences were evident for either group. Fathers in the control group had a significantly higher BMI (28.30, SD 9.09 kg/m^2^) compared with fathers in the intervention group (26.54, SD 3.76 kg/m^2^; *P*=.02). Among mothers, the highest proportion were unemployed and held a bachelor’s degree, although no significant differences in maternal demographic factors or BMI were observed between the control and intervention groups.

The main fruit goals chosen by the adolescents were starting the day with fruit or having fruit as a snack (29/55, 52%), having canned fruit or fruit as a sweet (25/55, 45%), and having fruit salad or fruit at dinner (16/55, 29%), whereas the least preferred goal was having fruit at lunch (8/55, 14%). Among the vegetable goals, the most frequently chosen goals were having vegetables at lunch (56%, 31/55), having bean salad (52%, 29/55), having dark green vegetables (47%, 26/55), having vegetables as a snack (36%, 20/55), starting the day with vegetables (34%,19/55), and having red or orange vegetables (32%,18/55), whereas the least chosen goal was having vegetables at dinner (30%,17/55).

**Table 1:** Baseline characteristics of the study participants in the control and intervention groups. Differences between the control and intervention groups were analyzed using the chi-square test.

			Control group (n=49)		Intervention group (n=55)		*P* value
Age (years), mean (SD)			15.57 (2)		14.74 (1)		.11
**Sex, n (%)**							<.001
	Adolescent boys	20 (41)		4 (7)			
	Adolescent girls	29 (59)		51 (93)			
**Type of school, n (%)**							.77
	Government	36 (73)		42 (76)			
	Private	13 (27)		13 (24)			
**BMI (kg/m^2^), n (%)**							.84
	Normal	40 (82)		45 (83)			
	Overweight	7 (14)		6 (11)			
	Obese	2 (4)		4 (6)			
**Paternal education, n (%)**							.31
	High school or lower	18 (36)		17 (32)			
	Bachelor	27 (55)		28 (51)			
	Postgraduate	4 (8)		10 (19)			
**Paternal occupation, n (%)**							.08
	Unemployed	1 (2)		3 (6)			
	Employed	42 (86)		37 (67)			
	Retired	6 (12)		15 (28)			
Paternal BMI (kg/m^2^), mean (SD)			28.30 (9)		26.54 (4)		.02
**Maternal occupation, n (%)**							.46
	Unemployed	28 (57)		35 (65)			
	Employed	18 (37)		19 (34)			
	Retired	2 (4)		1 (2)			
	Student	1 (2)		0			
**Maternal education, n (%)**							.37
	High school or lower	16 (33)		18 (33)			
	Bachelor	22 (45)		31 (56)			
	Postgraduate	11 (22)		6 (11)			
Maternal BMI (kg/m^2^), mean (SD)			26.55 (6)		26.41 (4)		.90
Number of siblings, mean (SD)			4.93 (2)		4.61 (2)		.50
**Family monthly income (SR^a^), n (%)**							.20
	Less than 5000	4 (8)		5 (9)			
	5000 to <10,000	4 (8)		13 (24)			
	10,000 to <20,000	21 (43)		22 (41)			
	>20,000	20 (41)		15 (28)			

^a^A conversion rate of 3.75 SR=US $1 applied.

### Effects of Using the Smartphone App on FV Scores

The baseline fruit consumption score in the intervention group (1.48, SD 0.99) was slightly higher than that in the control group (1.15, SD 0.68; Table 2). In the intervention group, no significant differences were observed between the scores obtained before and after the intervention for the consumption of either fruit (1.48, SD 0.99 and 1.70, SD 1.11, respectively; *P*=.31) or vegetables (1.50, SD 0.97 and 1.43, SD 1.03, respectively; *P*=.30). The control group showed a significant increase in fruit consumption scores between before (1.15, SD 0.68) and after the intervention (1.64, SD 0.98; *P*=.01), although it did not show a significant difference in vegetable consumption score (*P*=.54). However, no significant difference was observed between the intervention and control groups in fruit or vegetable consumption after the intervention.

The intake of individual fruit items increased in both groups (Multimedia Appendix 2). In the control group, there was a significant increase in the consumption of fruit juice (*P*<.001), fruit salad (*P*=.003), kiwi (*P*=.003), and pineapple (*P*=.01), whereas in the intervention group, there was a significant increase in the consumption of fruit juice (*P*=.02), grapefruit (*P*=.03), and guava (*P*=.02). No significant changes were observed in vegetable intake in the control group. However, there was a significant decrease in the consumption of potatoes (*P*=.003), lettuce (*P*=.007), and Jew’s mallow (*P*=.01) and an increase in the consumption of sweet potatoes (*P*=.02) in the intervention group. Moreover, a significant difference was found between the intervention and control groups after the intervention period (6 weeks) in some items, including grapefruit (*P*=.007), potatoes (*P*=.02), sweet potatoes (*P*=.04), carrots (*P*=.007), and cucumber (*P*=.007). Grapefruit and sweet potato intakes were higher in the intervention group after 6 weeks, whereas potato, carrot, and cucumber intakes were higher in the control group after 6 weeks.

**Table 2:** Fruit and vegetable scores in the control (n=49) and intervention (n=55) groups at baseline and after 6 weeks. The adjusted model was assessed using regression analysis. The estimates were adjusted for age, sex, the adolescents’ BMI, the parents’ education and BMI, and family income.

			Preintervention		Postintervention		Adjusted *P* value within group
**Fruit score, mean (SD)**							
	Control	1.15 (0.68)		1.64 (0.98)		.01	
	Intervention	1.48 (0.99)		1.70 (1.11)		.31	
	Adjusted *P* value between groups	.11		.52^a^		N/A^b^	
**Vegetable score, mean (SD)**							
	Control	1.44 (0.97)		1.55 (0.90)		.54	
	Intervention	1.50 (0.97)		1.43 (1.03)		.30	
	Adjusted *P* value between groups	.35		.35^a^		N/A	

^a^These estimates were also adjusted for preintervention values; these *P* values reflect the different effects of using the smartphone app between the control and intervention groups.

^b^N/A: not applicable.

## Discussion

### Principal Findings

The use of mobile phone–based approaches to encourage healthier lifestyles is becoming more common. To our knowledge, this is the first study conducted in Saudi Arabia to investigate the effects of using a smartphone app on FV intake among adolescents. Although the fruit consumption score was higher in the intervention group at baseline, this group showed no significant increase in fruit intake after 6 weeks of using the app, whereas the control group showed significantly higher fruit consumption. Both control and intervention groups showed no significant changes in vegetable consumption scores before or after the intervention. The control and intervention groups showed a significant increase in consumption of some fruit items, such as fruit juice, compared with the preintervention period. The intake of some vegetable items (ie, potatoes, lettuce, and Jew’s mallow) decreased in the intervention group after 6 weeks of using the app, whereas no significant changes in vegetable consumption were observed in the control group. Moreover, we found a significant difference in the consumption of some FV items between the control and intervention groups after 6 weeks of the intervention.

Consistent with our findings, several previous studies have demonstrated low FV consumption among school-aged children from different regions in Saudi Arabia. According to a previous cross-sectional study, only 12.8% and 22.8% of adolescents (n=2908, age 14-19 years) reported daily consumption of fruits and vegetables, respectively [23]. Similarly, two studies, one conducted among 1335 boys and the other among 512 girls, confirmed low FV intake (less than one portion per day) among teens in Saudi Arabia [9,24]. Additionally, data from 725 Saudi children (age 7-12 years) showed that 69% and 71.4%, respectively, did not consume FV daily [25]. These results highlight the importance of identifying approaches to promote FV consumption among young children and adolescents in Saudi Arabia. Furthermore, the prevalence of overweight and obesity reported in this study was relatively higher than that among Dutch adolescent boys (12.8% and 1.8%, respectively) and girls (14.8% and 2.2%, respectively) [26]. However, our study’s prevalence of overweight and obesity was similar to that reported among French adolescents (17.5% and 5.2%, respectively) [27]. Conversely, countries such as Greece have shown higher rates of overweight and obesity among children and adolescents, exceeding 35% [28].

In contrast to our findings, previous studies have confirmed the positive effects of technological interventions, including web-based platforms and smartphone apps, in promoting FV consumption. In one study, an innovative web-based platform (Team Nutriathlon) was used to promote FV intake among adolescents aged 13 to 14 years from the Quebec City region in Canada for 6 weeks. Team Nutriathlon is a 6-week school-based nutrition intervention. The website provided a 6-week calendar on which adolescents were asked to record their consumption of FVs from Monday to Friday. Team Nutriathlon increased FV consumption in the intervention group (n=193) by 3.4 servings, in comparison to an increase of 0.39 servings (n=89) in the control group [13]. Another study evaluated 154 university students (age 18-26 years) to assess the efficacy of web-based nutritional interventions and web interventions with daily text-messaging reminders to increase FV intake. The use of web interventions with daily text-messaging reminders for 4 weeks significantly increased vegetable intake, but not fruit intake [29]. A meta-analysis of the pooled results of 2 computer-based game interventions also showed increased FV consumption [30]. Even without the use of technology, interventions focused on promoting FV intake among children and adolescents showed a significant increase [31,32].

Indeed, it is crucial to note the role of parents in shaping eating behaviors and food preferences from an early stage. Parents are responsible for the availability of healthy food and encouraging children and adolescents to consume and accept different flavors, especially fruits and vegetables, as these foods are not very tasty [33]. Previous research indicates that children who are encouraged by their parents to consume and try fruits and vegetables in their early years have higher acceptance of these food items in their adolescence and adulthood [34]. Moreover, parental involvement in nutrition intervention targeting FV consumption among adolescents significantly improved adolescents’ intake of FV [35]. Furthermore, parents may provide positive role modeling to their children in the intake of healthy food. Previous studies showed that college students who dine away from their families and home are highly obese, as they consume foods high in fat and calories and low amounts of fruits and vegetables compared with their counterparts who eat with their families [36]. Thus, our study’s low consumption of FV among adolescents might be directly or indirectly related to their parents.

Several prior studies were conducted in schools with the involvement of teachers and peers, which had a positive effect on the adoption of healthy habits and increased FV consumption [37,38]. However, in the current study, the absence of support from teachers and peers due to the closure of schools as a result of the COVD-19 pandemic could be one of the reasons for the lack of a significant increase in FV consumption. The observed increase in fruit consumption in the control group in the current study could be explained by the Hawthorne effect [39], since the adolescents in this group were informed that there was a questionnaire to complete after the intervention period, which may have encouraged them to increase their consumption, especially after completing the baseline questionnaire (prior to the intervention). Moreover, in our study, we observed an increase in fruit juice intake in both the control and intervention groups. According to the National Health and Nutrition Examination Survey, fruit juice is the main contributor to fruit intake among US adolescents [40,41]. Nevertheless, during the study period, food shops were not closed, nor were there any restrictions placed on circulation that could have had an impact on family FV purchasing power. Moreover, given that the study was conducted between February and March (late winter and early spring), there were no seasonal differences affecting the availability of FV on the market.

### Limitations

Our study has several limitations, including a limited duration, which should be extended in future research. Second, measuring FV consumption using a self-administered questionnaire may have led to some limitations, although previous studies conducted among individuals in the same age group have employed the same questionnaire, and previous studies showed that a self-administered FFQ is an easy and useful tool for assessing dietary intake among adolescents [42,43]. Third, self-reported weight and height data are considered a limitation of this research; however, the research team instructed all participants on the appropriate methods for measuring weight and height, and a previous study confirmed that self-reporting weight and height is a valid method [44]. Fourth, the lack of involvement of parents in this intervention was also a limitation. However, our study has important strengths. The app is free, straightforward, and easily understood by adolescents, and a brochure explaining the use of the app in Arabic was provided to all adolescents in the intervention group. Moreover, our sample size had sufficient statistical power, and to our knowledge, this was the first study in Saudi Arabia that examined the effects of using a smartphone app to enhance FV intake among adolescents.

### Conclusions

The low FV intake among adolescents in Saudi Arabia highlights the importance of implementing an approach to promote FV intake in this population. However, the use of a smartphone app in the current study did not increase FV consumption among adolescents in the intervention group after 6 weeks. Future nutritional educational studies aiming to enhance the dietary patterns of adolescents should involve parents, as they have an important role in their children’s dietary patterns. Moreover, increased involvement by peers and teachers in schools can promote FV intake among adolescents with beneficial effects.

## Appendix

Multimedia Appendix 1 Supplementary Table 1. Fruit and vegetables listed in the food frequency questionnaire.

Multimedia Appendix 2 Supplementary Table 2. Comparison of fruit and vegetable item scores for the control (n=49) and intervention (n=55) groups at baseline and after 6 weeks.

Multimedia Appendix 3 CONSORT-eHEALTH checklist (V 1.6.2).

## Abbreviations

FFQ: food frequency questionnaire
FV: fruit and vegetable
