# Supplementary material for: Effects of a Smartphone App on Fruit and Vegetable Consumption Among Saudi Adolescents: Randomized Controlled Trial
Source: JMIR Pediatr Parent. 2023 Feb 9;6:e43160. doi: 10.2196/43160 (PMC9951076; doi:10.2196/43160)
Supplement: Multimedia Appendix 1 [file pediatrics_v6i1e43160_app1.docx]

**Supplementary Table 1.** Fruit and vegetables listed in the food frequency questionnaire

| **Vegetables (22 items)** | **Fruits (18 items)** |
| --- | --- |
| Potato | 100% fruit juice |
| Pumpkin | Fruit salad |
| Sweet potato | Dried fruit |
| Cauliflower | Apple |
| Green beans | Pear |
| Spinach | Orange |
| Cabbage | Mandarin |
| Peas | Grapefruit |
| Broccoli | Banana |
| Carrots | Peach |
| Zucchini | Plum or apricot |
| Eggplant | Mango |
| Sweet pepper | Guava |
| Corn | Kiwi |
| Mushrooms | Pineapple |
| Tomatoes | Grapes |
| Lettuce | Strawberries |
| Celery | Melon |
| Cucumber |  |
| Onion |  |
| Okra |  |
| Jew’s mallow (Arabic: mulukhiyah) |  |
