# Supplementary material for: Effects of a Smartphone App on Fruit and Vegetable Consumption Among Saudi Adolescents: Randomized Controlled Trial
Source: JMIR Pediatr Parent. 2023 Feb 9;6:e43160. doi: 10.2196/43160 (PMC9951076; doi:10.2196/43160)
Supplement: Multimedia Appendix 2 [file pediatrics_v6i1e43160_app2.docx]

**Supplementary Table 2.** Comparison of fruit and vegetable item scores for the control (n = 49) and intervention (n = 55) groups at baseline and after 6 weeks.

| ***P*** | **Post-intervention ^a^** | **Pre-intervention** | **Items** |
| --- | --- | --- | --- |
| **Fruit juice** | | | |
| <.001 | 2.45 ± 1.71 | 1.16 ± 0.89 | Control |
| .02 | 2.29 ± 1.55 | 1.54 ± 1.52 | Intervention |
|  | .53 | .22 | ***P*** |
| **Fruit salad** | | | |
| .003 | 1.86 ± 1.69 | 0.95 ± 1.33 | Control |
| .51 | 1.79 ± 1.48 | 1.50 ± 1.70 | Intervention |
|  | .80 | .68 | *P* |
| **Dried fruit** | | | |
| .049 | 0.83 ± 1.26 | 0.38 ± 0.72 | Control |
| .28 | 1.10 ± 1.53 | 0.69 ± 1.38 | Intervention |
|  | .75 | .86 | *P* |
| **Apple** | | | |
| .06 | 2.54 ± 1.58 | 1.92 ± 1.11 | Control |
| .68 | 2.18 ± 1.60 | 2.32 ± 1.58 | Intervention |
|  | .17 | .58 | *P* |
| **Orange** | | | |
| .18 | 2.55 ± 1.63 | 2.15 ± 1.35 | Control |
| .31 | 2.03 ± 1.34 | 2.14 ± 1.62 | Intervention |
|  | .12 | .45 | *P* |
| **Pear** | | | |
| .09 | 1.59 ± 1.50 | 0.97 ± 1.29 | Control |
| .25 | 1.29 ± 1.49 | 0.93 ± 1.38 | Intervention |
|  | .19 | .90 | *P* |
| **Mandarin** | | | |
| .61 | 2.13 ± 1.75 | 1.87 ± 1.20 | Control |
| .049 | 1.88 ± 1.42 | 2.39 ± 1.80 | Intervention |
|  | .43 | .17 | *P* |
| **Grapefruit** | | | |
| .43 | 0.52 ± 0.91 | 0.34 ± 0.77 | Control |
| .03 | 1.22 ± 1.49 | 0.65 ± 1.29 | Intervention |
|  | .007 | .71 | *P* |
| **Banana** | | | |
| .38 | 2.76 ± 1.75 | 2.51 ± 1.41 | Control |
| .85 | 2.09 ± 1.70 | 2.15 ± 1.64 | Intervention |
|  | .09 | .87 | *P* |
| **Peach** | | | |
| .58 | 1.04 ± 1.30 | 0.78 ± 1.23 | Control |
| .61 | 1.27 ± 1.49 | 1.05 ± 1.66 | Intervention |
|  | .78 | .27 | *P* |
| **Apricot** | | | |
| .73 | 0.87 ± 1.16 | 0.70 ± 1.14 | Control |
| .17 | 1.31 ± 1.58 | 0.94 ± 1.43 | Intervention |
|  | .56 | .43 | *P* |
| **Mango** | | | |
| .90 | 1.28 ± 1.14 | 1.23 ± 1.34 | Control |
| .48 | 1.76 ± 1.51 | 1.53 ± 1.67 | Intervention |
|  | .29 | .73 | *P* |
| **Guava** | | | |
| .06 | 1.26 ± 1.35 | 0.71 ± 1.13 | Control |
| .02 | 1.40 ± 1.56 | 0.76 ± 1.32 | Intervention |
|  | .83 | .65 | *P* |
| **Kiwi** | | | |
| .003 | 1.35 ± 1.47 | 0.54 ± 0.92 | Control |
| .17 | 1.69 ± 1.74 | 1.17 ± 1.63 | Intervention |
|  | .74 | .08 | *P* |
| **Pineapple** | | | |
| .01 | 1.28 ± 1.47 | 0.60 ± 0.89 | Control |
| .17 | 1.63 ± 1.36 | 1.29 ± 1.41 | Intervention |
|  | .59 | .08 | *P* |
| **Grape** | | | |
| .06 | 1.94 ± 1.66 | 1.30 ± 1.21 | Control |
| .25 | 1.83 ± 1.61 | 2.12± 1.69 | Intervention |
|  | .91 | .049 | *P* |
| **Strawberry** | | | |
| .53 | 1.91 ± 1.63 | 1.66 ± 1.08 | Control |
| .45 | 1.77 ± 1.51 | 2.05 ± 1.62 | Intervention |
|  | .23 | .67 | *P* |
| **Cherry** | | | |
| .19 | 1.37 ± 1.57 | 0.89 ± 1.19 | Control |
| .07 | 1.90 ± 1.86 | 1.45 ± 1.90 | Intervention |
|  | .35 | .47 | *P* |
| **Potato** | | | |
| .56 | 2.86 ± 1.66 | 2.65 ± 1.81 | Control |
| .003 | 2.28 ± 1.46 | 2.88 ± 1.74 | Intervention |
|  | .02 | .21 | *P* |
| **Pumpkin** | | | |
| .82 | 0.39 ± 0.92 | 0.35 ± 0.84 | Control |
| .27 | 0.78 ± 1.09 | 0.52 ± 0.95 | Intervention |
|  | .14 | .95 | *P* |
| **Sweet potato** | | | |
| .63 | 0.63 ± 1.21 | 0.74 ± 1.27 | Control |
| .02 | 1.07 ± 1.38 | 0.54 ± 1.22 | Intervention |
|  | .04 | .81 | *P* |
| **Cauliflower** | | | |
| .64 | 0.95 ± 1.36 | 0.86 ± 1.34 | Control |
| .12 | 1.21 ± 1.47 | 0.72 ± 1.33 | Intervention |
|  | .12 | .53 | *P* |
| **Green bean** | | | |
| .57 | 1.46 ± 1.45 | 1.31 ± 1.43 | Control |
| .54 | 1.40 ± 1.47 | 1.12 ± 1.65 | Intervention |
|  | .51 | .54 | *P* |
| **Spinach** | | | |
| .48 | 0.96 ± 1.30 | 0.81 ± 1.10 | Control |
| .22 | 1.26 ± 1.79 | 0.83 ± 1.52 | Intervention |
|  | .76 | .18 | *P* |
| **Cabbage** | | | |
| .59 | 1.15 ± 1.55 | 1.28 ± 1.38 | Control |
| .51 | 1.20 ± 1.42 | 1.18 ± 1.38 | Intervention |
|  | .88 | .94 | *P* |
| **Red kidney beans** | | | |
| .86 | 0.80 ± 0.91 | 0.77 ± 1.00 | Control |
| .52 | 1.17 ± 1.59 | 0.89 ± 1.46 | Intervention |
|  | .43 | .99 | *P* |
| **Broccoli** | | | |
| .50 | 0.86 ± 1.20 | 0.70 ± 1.16 | Control |
| .77 | 0.92 ± 1.30 | 0.71 ± 1.33 | Intervention |
|  | .52 | .64 | *P* |
| **Carrots** | | | |
| .43 | 2.37 ± 1.58 | 2.06 ± 1.79 | Control |
| .049 | 1.62 ± 1.48 | 1.98 ± 1.73 | Intervention |
|  | .007 | .63 | *P* |
| **Zucchini** | | | |
| .78 | 1.27 ± 1.62 | 1.41 ± 1.59 | Control |
| .37 | 1.19 ± 1.38 | 1.21 ± 1.40 | Intervention |
|  | .68 | .39 | *P* |
| **Eggplant** | | | |
| .88 | 1.24 ± 1.37 | 1.28 ± 1.44 | Control |
| .92 | 1.28 ± 1.55 | 1.51 ± 1.60 | Intervention |
|  | .92 | .82 | *P* |
| **Sweet pepper** | | | |
| .52 | 1.77 ± 1.61 | 1.61 ± 1.58 | Control |
| .91 | 1.65 ± 1.47 | 1.59 ± 1.57 | Intervention |
|  | .80 | .24 | *P* |
| **Corn** | | | |
| .71 | 1.76 ± 1.57 | 1.64 ± 1.38 | Control |
| .21 | 1.70 ± 1.31 | 2.20 ± 1.89 | Intervention |
|  | .74 | .04 | *P* |
|  |  |  | **Mushroom** |
| .96 | 0.82 ± 1.19 | 0.79 ± 1.36 | Control |
| .39 | 1.14 ± 1.58 | 0.90 ± 1.66 | Intervention |
|  | .15 | .24 | *P* |
| **Tomato** | | | |
| .76 | 2.62 ± 1.69 | 2.55 ± 2.13 | Control |
| .18 | 2.09 ± 1.59 | 2.62 ± 2.30 | Intervention |
|  | .15 | .70 | *P* |
| **Lettuce** | | | |
| .26 | 2.56 ± 1.64 | 2.25 ± 1.36 | Control |
| .007 | 1.96 ± 1.51 | 2.80 ± 2.03 | Intervention |
|  | .10 | .12 | *P* |
| **Cucumber** | | | |
| .26 | 3.21 ± 1.84 | 2.78 ± 1.65 | Control |
| .12 | 2.27 ± 1.53 | 2.70 ± 2.04 | Intervention |
|  | .007 | .85 | *P* |
| **Celery** | | | |
| .88 | 0.62 ± 1.09 | 0.59 ± 1.05 | Control |
| .89 | 0.82 ± 1.33 | 0.76 ± 1.49 | Intervention |
|  | .48 | .44 | *P* |
| **Onion** | | | |
| .33 | 2.67 ± 2.07 | 2.38 ± 1.75 | Control |
| .62 | 2.14 ± 1.51 | 2.18 ± 1.51 | Intervention |
|  | .91 | .73 | *P* |
| **Okra** | | | |
| .81 | 1.50 ± 1.30 | 1.41 ± 1.43 | Control |
| .08 | 1.06± 1.25 | 1.33± 1.65 | Intervention |
|  | .14 | .40 | *P* |
| **Mallow** | | | |
| .63 | 1.66 ± 1.32 | 1.54 ± 1.28 | Control |
| 001 | 1.36 ± 1.26 | 2.20 ± 2.91 | Intervention |
|  | .12 | .17 | P |

The adjusted model was assessed using regression analysis. The estimates were adjusted for age, sex, adolescents' body mass index, parents' education and body mass index, and family income.

a P values, presented in column post-intervention, reflect the effect of using smartphone application between the control and intervention groups.
