# Supplementary material for: Effects of a Smartphone App on Fruit and Vegetable Consumption Among Saudi Adolescents: Randomized Controlled Trial
Source: JMIR Pediatr Parent. 2023 Feb 9;6:e43160. doi: 10.2196/43160 (PMC9951076; doi:10.2196/43160)
Supplement: Multimedia Appendix 3 [file pediatrics_v6i1e43160_app3.pdf]

CONSORT-EHEALTH Checklist V1.6.2 Report	Manuscript Number	43160
**(based on CONSORT-EHEALTH V1.6), available at [http://tinyurl.com/consort-ehealth-v1-6].**		
**Date completed**		
1/5/2023 5:33:50		
**by**		
Israa Shatwan		
Effects of a Smartphone Application on Fruit and Vegetable Consumption Among Saudi Adolescents: A Randomized Intervention Study		
TITLE		
**1a-i) Identify the mode of delivery in the title**		
"Smartphone Application "		
**1a-ii) Non-web-based components or important co-interventions in title**		
**1a-iii) Primary condition or target group in the title**		
"Effects of a Smartphone Application on Fruit and Vegetable Consumption Among Saudi Adolescents: A Randomized Intervention Study"		
ABSTRACT		
**1b-i) Key features/functionalities/components of the intervention and comparator in the METHODS section of the ABSTRACT**		
"We examined the effects of a smartphone application (“My Plate”) on fruit and vegetable intake over 6 weeks in the intervention group. Pre- and postintervention questionnaires were used in the intervention and control groups."		
**1b-ii) Level of human involvement in the METHODS section of the ABSTRACT**		
**1b-iii) Open vs. closed, web-based (self-assessment) vs. face-to-face assessments in the METHODS section of the ABSTRACT**		
**1b-iv) RESULTS section in abstract must contain use data**		
**1b-v) CONCLUSIONS/DISCUSSION in abstract for negative trials**		
"These findings suggest that a smartphone application did not significantly improve fruit and vegetable intake among adolescents. "		
INTRODUCTION		
**2a-i) Problem and the type of system/solution**		
"F&V consumption among adolescents does not meet these recommendations. For instance, the UK National Diet and Nutrition Survey found that consumption of fruits among adolescents declined compared with that in early childhood, even though vegetable consumption showed no change [7]. In Saudi Arabia, most adolescents reported that they consume low amounts F&Vs daily [8,9]. Education and knowledge about food and nutrition are among the factors that influence food choices [6]."<br>"Recently, smartphone applications have been used to promote health and wellness among individuals in various communities. As a result, several nutritional interventions have been attempted to determine the usefulness of these applications in promoting healthy dietary habits. One of the advantages of these applications is that they can serve as cost-effective and flexible platforms for implementing behavioral changes in nutrition"		
**2a-ii) Scientific background, rationale: What is known about the (type of) system**		
"A diet rich in F&Vs is associated with numerous health benefits. F&Vs are a good source of vitamins A and C, minerals, electrolytes, phytochemicals, antioxidants, and dietary fiber [6]. The World Health Organization recommends F&V intake amounting to ≥400 g/day to maintain optimal health, reduce the risk of non-communicable diseases, including heart disease, cancer, type II diabetes mellitus, and obesity, and to prevent micronutrient deficiencies [6].<br>In Saudi Arabia, most adolescents reported that they consume low amounts F&Vs daily [8,9]. "		
**Does your paper address CONSORT subitem 2b?**		
"In this study, we aimed to examine the effects of the “MyPlate” smartphone application on F&V intake over 6 weeks in adolescents from Jeddah, Saudi Arabia."		
METHODS		
**3a) CONSORT: Description of trial design (such as parallel, factorial) including allocation ratio**		
"This was a randomized intervention study conducted on 104 adolescents: 49 in the control group and 55 in the intervention group. In total, 146 adolescents were initially recruited, of which 26 withdrew from the study because they did not complete the baseline questionnaire or voluntarily decided to withdraw. The remaining 120 adolescents were randomly divided into intervention and control groups. Microsoft Excel (version 22, Microsoft Corp., Redmond, WA, USA) with the RAND function was used to randomize the sample. After generating a random number, the participants were divided into control and intervention groups. As 16 adolescents decided not to complete the study or did not fill in the final questionnaire, 104 adolescents (24 boys and 80 girls) completed the intervention phase of the study (Figure 1). "		
**3b) CONSORT: Important changes to methods after trial commencement (such as eligibility criteria), with reasons**		
"The inclusion criteria were healthy boys and girls (based on the self-reported absence of diseases, such as diabetes mellitus, that may influence food intake) aged 13–18 years, at schools, and able to use smartphones, either their own or their parents. The exclusion criteria were unhealthy adolescents or those not within the age range of this study. "		
**3b-i) Bug fixes, Downtimes, Content Changes**		
**4a) CONSORT: Eligibility criteria for participants**		
"The inclusion criteria were healthy boys and girls (based on the self-reported absence of diseases, such as diabetes mellitus, that may influence food intake) aged 13–18 years, at schools, and able to use smartphones, either their own or their parents."		
**4a-i) Computer / Internet literacy**		
"The inclusion criteria were healthy boys and girls aged 13–18 years, at schools, and able to use smartphones"		
**4a-ii) Open vs. closed, web-based vs. face-to-face assessments:**		
"Since schools were closed because of the coronavirus disease 2019 pandemic, adolescents were recruited through invitations sent via emails and WhatsApp application to their parents using snowballing recruitment. The invitation to the study was first sent to members and Bachelor of Science students of the Food and Nutrition Department at King Abdulaziz University to help the research team with recruitment. Then, the research team contacted the parents and adolescents who voluntarily agreed to participate in the study. "		
**4a-iii) Information giving during recruitment**		
"The study procedures were explained to the parents or guardians of all prospective participants. All adolescents were voluntarily recruited and provided verbal consent for participation in the study; their parents or guardians provided written informed consent. "		
**4b) CONSORT: Settings and locations where the data were collected**		
"Adolescents in the intervention group were divided into 11 smaller groups, each containing five participants to explain the application. One of the researchers conducted video conference calls with each of these small intervention groups. The researcher provided a brief presentation about the health benefits and appropriate serving sizes of F&Vs and explained how to use the smartphone application.<br>All measures were collected via an online questionnaire on Google Forms. The questionnaire consisted of two parts. The first part included sociodemographic data, including information regarding age, sex, school type (private or public), weight and height of the adolescents and their parents, parents’ education level (high school or lower, bachelor’s degree, or postgraduate degree), parents’ occupation (employed or unemployed), number of children in the family, and family income. Second part was food frequency questionnaire"		
**4b-i) Report if outcomes were (self-)assessed through online questionnaires**		
"All measures were collected via an online questionnaire on Google Forms. The questionnaire consisted of two parts. The first part included sociodemographic data, including information regarding age, sex, school type (private or public), weight and height of the adolescents and their parents, parents’ education level (high school or lower, bachelor’s degree, or postgraduate degree), parents’ occupation (employed or unemployed), number of children in the family, and family income. "		
**4b-ii) Report how institutional affiliations are displayed**		
**5) CONSORT: Describe the interventions for each group with sufficient details to allow replication, including how and when they were actually administered**		
**5-i) Mention names, credential, affiliations of the developers, sponsors, and owners**		
"This study used the free nutritional “MyPlate” smartphone application which is readily available on both iOS and Android platforms to promote F&V intake among adolescents. The application is a multi-component communications plan that was developed by the US Department of Agriculture Food and Nutrition Service in 2011. The app aids in translating the American Dietary Guidelines to the public and can be used as a nutritional education resource for children and adults. The application icon is an easy, effective, visual platform that helps promote healthy food choices, including all food groups, and create a balanced plate at mealtimes. The application allows one to set daily healthy eating goals for each food group and track individual progress [15,16]."		
**5-ii) Describe the history/development process**		
**5-iii) Revisions and updating**		

**5-iv) Quality assurance methods**		
**5-v) Ensure replicability by publishing the source code, and/or providing screenshots/screen-capture video, and/or providing flowcharts of the algorithms used**		
**5-vi) Digital preservation**		
**5-vii) Access**		
"They were encouraged to turn on notifications for the application to receive reminder messages. The research team also sent weekly WhatsApp text message reminders (in Arabic language) to the adolescents. The intervention period was 6 weeks. Adolescents in the control group were not exposed to the smartphone application and did not receive any advice to promote their F&V consumption, which may have affected their F&V consumption"		
**5-viii) Mode of delivery, features/functionalities/components of the intervention and comparator, and the theoretical framework**		
behavioral change intervention		
**5-ix) Describe use parameters**		
"One of the researchers conducted video conference calls with each of these small intervention groups. The researcher provided a brief presentation about the health benefits and appropriate serving sizes of F&Vs and explained how to use the smartphone application. An instructional brochure was provided in Arabic to all adolescents in the intervention group. The research team was available to assist participants at any time during the study period. The participants were required to choose three of seven goals for each F&V. The fruit goals were as follows: (1) have fruit with dinner; (2) add fruit to your salad; (3) snack on fruit; (4) have fruit for a sweet treat; (5) have fruit with lunch; (6) add frozen, canned, or dried fruit to your meal; and (7) start your day with fruit. The vegetable goals were as follows: (1) have vegetables with dinner; (2) have a dark green vegetable; (3) start your day with vegetables; (4) have a red or orange vegetable; (5) have vegetables with lunch; (6) snack on vegetables; and (7) make a salad or side dish using beans, peas, or lentils. After choosing six goals (three for fruit and three for vegetables), the participants were required to mark the goal that they chose daily and were requested to adhere to their chosen goals until the end of the study. They were encouraged to turn on notifications for the application to receive reminder messages. The research team also sent weekly WhatsApp text message reminders (in Arabic language) to the adolescents. The intervention period was 6 weeks. Adolescents in the control group were not exposed to the smartphone application and did not receive any advice to promote their F&V consumption, which may have affected their F&V consumption. Instead, they were only asked to complete the pre- and post-questionnaires. "		
**5-x) Clarify the level of human involvement**		
"One of the researchers conducted video conference calls with each of these small intervention groups. The researcher provided a brief presentation about the health benefits and appropriate serving sizes of F&Vs and explained how to use the smartphone application. An instructional brochure was provided in Arabic to all adolescents in the intervention group. The research team was available to assist participants at any time during the study period. "		
**5-xi) Report any prompts/reminders used**		
"They were encouraged to turn on notifications for the application to receive reminder messages. The research team also sent weekly WhatsApp text message reminders (in Arabic language) to the adolescents. "		
**5-xii) Describe any co-interventions (incl. training/support)**		
"Adolescents in the intervention group were divided into 11 smaller groups, each containing five participants to explain the application. One of the researchers conducted video conference calls with each of these small intervention groups. The researcher provided a brief presentation about the health benefits and appropriate serving sizes of F&Vs and explained how to use the smartphone application. An instructional brochure was provided in Arabic to all adolescents in the intervention group. The research team was available to assist participants at any time during the study period. "		
**6a) CONSORT: Completely defined pre-specified primary and secondary outcome measures, including how and when they were assessed**		
"The intervention period was 6 weeks. Adolescents in the control group were not exposed to the smartphone application and did not receive any advice to promote their F&V consumption, which may have affected their F&V consumption. Instead, they were only asked to complete the pre- and post-questionnaires. "		
**6a-i) Online questionnaires: describe if they were validated for online use and apply CHERRIES items to describe how the questionnaires were designed/deployed**		
"Second, measuring F&V consumption using a self-administered questionnaire may have led to some limitations, although previous studies conducted among individuals in the same age group have employed the same questionnaire, and previous studies showed that a self-administered FFQ is an easy and useful tool for assessing dietary intake among adolescents [41,42]. Third, self-reported weight and height data are considered a limitation of this research; however, the research team instructed all participants on the appropriate methods for measuring weight and height, and a previous study confirmed that self-reporting weight and height is a valid method [43]. "		
**6a-ii) Describe whether and how "use" (including intensity of use/dosage) was defined/measured/monitored**		
**6a-iii) Describe whether, how, and when qualitative feedback from participants was obtained**		
After six weeks questionnaire were collected again from participants		
**6b) CONSORT: Any changes to trial outcomes after the trial commenced, with reasons**		
"Adolescents in the intervention group were divided into 11 smaller groups, each containing five participants to explain the application. One of the researchers conducted video conference calls with each of these small intervention groups. The researcher provided a brief presentation about the health benefits and appropriate serving sizes of F&Vs and explained how to use the smartphone application.<br>All measures were collected via an online questionnaire on Google Forms. The questionnaire consisted of two parts. The first part included sociodemographic data, including information regarding age, sex, school type (private or public), weight and height of the adolescents and their parents, parents' education level (high school or lower, bachelor's degree, or postgraduate degree), parents' occupation (employed or unemployed), number of children in the family, and family income. Second part was food frequency questionnaire"		
**7a) CONSORT: How sample size was determined**		
**7a-i) Describe whether and how expected attrition was taken into account when calculating the sample size**		
"The sample size was determined based on the ability to detect an expected mean difference of .7 servings according to a previous parallel intervention study [18] with 80% power and a 5% significance level. The calculated required sample size was 33 adolescents in each group [19]. Thus, considering a nearly 50% drop-out rate, we recruited 60 participants for each group. "		
**7b) CONSORT: When applicable, explanation of any interim analyses and stopping guidelines**		
"The intervention period was 6 weeks. Adolescents in the control group were not exposed to the smartphone application and did not receive any advice to promote their F&V consumption, which may have affected their F&V consumption. Instead, they were only asked to complete the pre- and post-questionnaires. "		
**8a) CONSORT: Method used to generate the random allocation sequence**		
"The remaining 120 adolescents were randomly divided into intervention and control groups. Microsoft Excel (version 22, Microsoft Corp., Redmond, WA, USA) with the RAND function was used to randomize the sample. After generating a random number, the participants were divided into control and intervention groups."		
**8b) CONSORT: Type of randomisation; details of any restriction (such as blocking and block size)**		
"The remaining 120 adolescents were randomly divided into intervention and control groups. Microsoft Excel (version 22, Microsoft Corp., Redmond, WA, USA) with the RAND function was used to randomize the sample. After generating a random number, the participants were divided into control and intervention groups."		
**9) CONSORT: Mechanism used to implement the random allocation sequence (such as sequentially numbered containers), describing any steps taken to conceal the sequence until interventions were assigned**		
"The remaining 120 adolescents were randomly divided into intervention and control groups. Microsoft Excel (version 22, Microsoft Corp., Redmond, WA, USA) with the RAND function was used to randomize the sample. After generating a random number, the participants were divided into control and intervention groups."		
**10) CONSORT: Who generated the random allocation sequence, who enrolled participants, and who assigned participants to interventions**		
"The remaining 120 adolescents were randomly divided into intervention and control groups. Microsoft Excel (version 22, Microsoft Corp., Redmond, WA, USA) with the RAND function was used to randomize the sample. After generating a random number, the participants were divided into control and intervention groups."		
**11a) CONSORT: Blinding - If done, who was blinded after assignment to interventions (for example, participants, care providers, those assessing outcomes) and how**		
**11a-i) Specify who was blinded, and who wasn't**		
There were no blinded in this study because participants use the application they knows that		
**11a-ii) Discuss e.g., whether participants knew which intervention was the "intervention of interest" and which one was the "comparator"**		
**11b) CONSORT: If relevant, description of the similarity of interventions**		
this is not applicable to the current study		
**12a) CONSORT: Statistical methods used to compare groups for primary and secondary outcomes**		
"Changes in F&V scores between baseline and after the intervention period in the control and intervention groups were analyzed using univariate regression. The smartphone application's effectiveness was examined by comparing F&V scores between intervention and control groups at 6 weeks through univariate regression. Univariate linear models were adjusted for age, sex, parental education, family income, adolescent and parental BMI, and baseline values in the analysis to test the effects of the smartphone application. The data are reported as means ± standard deviations."		
**12a-i) Imputation techniques to deal with attrition / missing values**		

this is not applicable to the current study

12b) CONSORT: Methods for additional analyses, such as subgroup analyses and adjusted analyses

this is not applicable to the current study

RESULTS

13a) CONSORT: For each group, the numbers of participants who were randomly assigned, received intended treatment, and were analysed for the primary outcome

"In total, 104 adolescents completed the study, of whom 23.1% were boys (24 out of 104 adolescents). The mean age of the adolescents was 15.1 ± 1.6 years, and 75.0% (78 out of 104 adolescents) attended public schools. The mean weight was 56.9 ± 15.8 kg. Most of the adolescents were in the normal body weight range, whereas 10.6% (13 out of 104 adolescents) were overweight, and 4.8% (6 out of 104 adolescents) were obese (Table 1). The majority of parents held a bachelor's as their highest degree. Most fathers were employed whereas mothers were unemployed. Most families had a middle-high income level (household income of 10,000 to 20,000 Saudi Riyal). The proportion of boys compared with girls was lower in the intervention group. No other significant baseline differences were evident for either group. Fathers in the control group had a significantly higher BMI (28.30 ± 9.09 kg/m2) compared with fathers in the intervention group (26.54 ± 3.76 kg/m2; P=.02). Among mothers, the highest percentages were unemployed and held bachelor's degrees, although no significant differences in maternal demographic factors and BMI were observed between control and intervention groups. "

13b) CONSORT: For each group, losses and exclusions after randomisation, together with reasons

Yes figure 1 shows CONSOPT flow diagram

13b-i) Attrition diagram

14a) CONSORT: Dates defining the periods of recruitment and follow-up

"This randomized intervention study was conducted between February and March 2021 among adolescents from Jeddah
The study period was 6 weeks"

14a-i) Indicate if critical “secular events” fell into the study period

14b) CONSORT: Why the trial ended or was stopped (early)

This is not applicable to current study. The study end after 6 weeks period of study after collecting questionnaire from participants

15) CONSORT: A table showing baseline demographic and clinical characteristics for each group

"Table 1. Baseline characteristics of the study participants in the control and intervention groups."

15-i) Report demographics associated with digital divide issues

"Table 1. Baseline characteristics of the study participants in the control and intervention groups."

16a) CONSORT: For each group, number of participants (denominator) included in each analysis and whether the analysis was by original assigned groups

16-i) Report multiple “denominators” and provide definitions

This is not applicable to the current study

16-ii) Primary analysis should be intent-to-treat

"The baseline fruit consumption score in the intervention group (1.48 ± .99) was slightly higher than that in the control group (1.15 ± .68) (Table 2). In the intervention group, no significant differences were observed between the scores obtained before and after the intervention for the consumption of either fruit (1.48 ± .99 and 1.70 ± 1.11, respectively; P=.31) or vegetables (1.50 ± .97 and 1.43 ± 1.03, respectively; P=.30). The control group showed a significant increase in fruit consumption scores between before (1.15 ± .68) and after the intervention (1.64 ± .98; P=.01), although it did not show a significant difference in vegetable consumption score (P=.54). However, no significant difference was observed between the intervention and control groups in fruit or vegetable consumption after the intervention. "

17a) CONSORT: For each primary and secondary outcome, results for each group, and the estimated effect size and its precision (such as 95% confidence interval)

Not included in the current study

17a-i) Presentation of process outcomes such as metrics of use and intensity of use

17b) CONSORT: For binary outcomes, presentation of both absolute and relative effect sizes is recommended

Not included in the current study the data were continues variable

18) CONSORT: Results of any other analyses performed, including subgroup analyses and adjusted analyses, distinguishing pre-specified from exploratory

Not included in the current study

18-i) Subgroup analysis of comparing only users

19) CONSORT: All important harms or unintended effects in each group

Not applicable in the current study

19-i) Include privacy breaches, technical problems

19-ii) Include qualitative feedback from participants or observations from staff/researchers

DISCUSSION

20) CONSORT: Trial limitations, addressing sources of potential bias, imprecision, multiplicity of analyses

20-i) Typical limitations in ehealth trials

"Our study has several limitations, including a limited duration, which should be extended in future research. Second, measuring F&V consumption using a self-administered questionnaire may have led to some limitations, although previous studies conducted among individuals in the same age group have employed the same questionnaire, and previous studies showed that a self-administered FFQ is an easy and useful tool for assessing dietary intake among adolescents [41,42]. Third, self-reported weight and height data are considered a limitation of this research; however, the research team instructed all participants on the appropriate methods for measuring weight and height, and a previous study confirmed that self-reporting weight and height is a valid method [43]. Fourth, the lack of involvement of parents in this intervention was also a limitation. However, our study has important strengths. The application is free, straightforward, and easily understood by adolescents, and a brochure explaining the use of the application in Arabic was provided to all adolescents in the intervention group. Moreover, our sample size had sufficient statistical power, and to our knowledge, this was the first study in Saudi Arabia that examined the effects of using a smartphone application to enhance F&V intake among adolescents. "

21) CONSORT: Generalisability (external validity, applicability) of the trial findings

21-i) Generalizability to other populations

21-ii) Discuss if there were elements in the RCT that would be different in a routine application setting

22) CONSORT: Interpretation consistent with results, balancing benefits and harms, and considering other relevant evidence

22-i) Restate study questions and summarize the answers suggested by the data, starting with primary outcomes and process outcomes (use)

"The use of mobile phone-based approaches to encourage healthier lifestyles is becoming more common. To our knowledge, this is the first study conducted in Saudi Arabia to investigate the effects of using a smartphone application on F&V intake among adolescents. Although the fruit consumption score was higher in the intervention group at baseline, this group showed no significant increase in fruit intake after 6 weeks of using the application, whereas the control group showed significantly higher fruit consumption. Both control and intervention groups showed no significant changes in vegetable consumption scores before or after the intervention. The control and intervention groups showed a significant increase in consumption of some fruit items, such as fruit juice, compared with the pre-intervention period. The intake of some vegetable items (ie, potatoes, lettuce, and Jew’s mallow) decreased in the intervention group after 6 weeks of using the application, whereas no significant changes in vegetable consumption were observed in the control group. Moreover, we found a significant difference in the consumption of some F&V items between the control and intervention groups after 6 weeks of the intervention."

22-ii) Highlight unanswered new questions, suggest future research

"Future nutritional educational studies aimed to enhance the dietary patterns of adolescents should involve parents as they have an important role in their children's dietary patterns. Moreover, increased involvement by peers and teachers in schools can promote F&V intake among adolescents with beneficial effects."

Other information

23) CONSORT: Registration number and name of trial registry

"Study procedures were approved by the Unit of Biomedical Ethics Research Committee at King Abdulaziz University (reference number: 101-21).'
This study not RCT, it nutrition education intervention

24) CONSORT: Where the full trial protocol can be accessed, if available

"Study procedures were approved by the Unit of Biomedical Ethics Research Committee at King Abdulaziz University (reference number: 101-21)."

25) CONSORT: Sources of funding and other support (such as supply of drugs), role of funders

Not applicable in the current study

X26-i) Comment on ethics committee approval

"Study procedures were approved by the Unit of Biomedical Ethics Research Committee at King Abdulaziz University (reference number: 101-21)."

**x26-ii) Outline informed consent procedures**		
"All adolescents were voluntarily recruited and provided verbal consent for participation in the study; their parents or guardians provided written informed consent. "		
**X26-iii) Safety and security procedures**		
**X27-i) State the relation of the study team towards the system being evaluated**		
"Conflicts of Interest<br>None declared."		
